# Enrichment of human embryonic stem cell-derived V3 interneurons using an *Nkx2-2* gene-specific reporter

**DOI:** 10.1038/s41598-023-29165-z

**Published:** 2023-02-03

**Authors:** Ieva Berzanskyte, Federica Riccio, Carolina Barcellos Machado, Elizabeth J. Bradbury, Ivo Lieberam

**Affiliations:** 1 Centre for Gene Therapy and Regenerative Medicine, Centre for Developmental Neurobiology, MRC Centre for Neurodevelopmental Disorders, King’s College London, 28th Floor Tower Wing, Guy’s Campus, Great Maze Pond, London, SE1 9RT UK; 2 The Wolfson Centre for Age-Related Diseases, King’s College London, London, UK

**Keywords:** Cell biology, Stem cells, Isolation, separation and purification

## Abstract

V3 spinal interneurons are a key element of the spinal circuits, which control motor function. However, to date, there are no effective ways of deriving a pure V3 population from human pluripotent stem cells. Here, we report a method for differentiation and isolation of spinal V3 interneurons, combining extrinsic factor-mediated differentiation and magnetic activated cell sorting. We found that differentiation of V3 progenitors can be enhanced with a higher concentration of Sonic Hedgehog agonist, as well as culturing cells in 3D format. To enable V3 progenitor purification from mixed differentiation cultures, we developed a transgene reporter, with a part of the regulatory region of V3-specific gene *Nkx2-2* driving the expression of a membrane marker CD14. We found that in human cells, NKX2-2 initially exhibited co-labelling with motor neuron progenitor marker, but V3 specificity emerged as the differentiation culture progressed. At these later differentiation timepoints, we were able to enrich V3 progenitors labelled with CD14 to ~ 95% purity, and mature them to postmitotic V3 interneurons. This purification tool for V3 interneurons will be useful for in vitro disease modeling, studies of normal human neural development and potential cell therapies for disorders of the spinal cord.

## Introduction

Ventral spinal interneurons are an integral part of spinal neural circuits—they integrate and relay information received locally and from supraspinal projections. Recent studies have shed light on their importance in generating central pattern generator (CPG) rhythmic outputs^1–5^ and fine-tuning the motor output^6,7^. Moreover, propriospinal interneurons have been shown to be actively recruited into newly forming circuits after spinal cord injury, and therefore, they have an important role in neuroplasticity^8,9^. These findings suggest that interneurons should be a key component of in vitro models of spinal neural circuits and represent a promising candidate for network restoration in disease or injury to the spinal cord.

Interneuron-specific transplantation studies for spinal cord injury treatment have previously been performed using the subclass V2a with promising histological and functional recovery outcomes^10,11^. However, the use of spinal interneurons in transplantation, as well as in vitro modelling, is restricted by the scarcity of these cell types in embryonic spinal cord tissue and by the complex cell type composition of ex vivo cell preparations. Recently, emerging stem cell differentiation protocols have offered a breakthrough in using specific interneuron subtypes. Mouse embryonic stem cells (mESCs) have been used to derive V2a interneurons^12^, V3 interneurons^6,13^, and most recently V0 interneurons^14^. However, successful optimised differentiation of human pluripotent stem cells (PSCs) into specific interneuron subtypes, to date, has only been achieved for V2a interneurons^15,16^.

V3 interneurons are one of the key populations considered for spinal cord injury therapy because they are involved in locomotion—behaviours such as swimming, runnning and walking^17,18^, and especially in establishing the regularity of locomotor rhythm^19^. In vitro, V3 and V2a interneurons alone are enough to induce rhythmic bursts similar to drug–induced fictive CPG activity^20^. They have an advantage of being largely commissural (80% of the cells) and projecting caudally—playing a significant role in contralateral excitation^21,22^, as well as being connected to corticospinal tract projections in a healthy spinal cord^23^. V3 interneurons are interconnected with different spinal neuron subtypes: they provide 22% of the vesicular glutamate transporter 2 (vGlut2+) synapses onto motor neurons, 24–27% of the vGlut2+ contacts onto inhibitory interneurons, and the rest connect onto LIM Homeobox 3 (Lhx3+) V2 interneurons and other commissural interneurons^21^. These qualities relating to extensive connectivity and functional involvement in locomotion make V3 interneurons a good target for cell transplantation. However, the lack of human derivation strategies for this spinal interneuron subtype^24^ represents a severe limitation for the advancement of the fields of in vitro modelling and development of cell therapies for spinal cord injury.

In this report, we describe a combined strategy for derivation and purification of V3 spinal interneurons from a human embryonic stem cell (hESC) model. We enriched for NKX2-2-expressing V3 progenitors in differentiation cultures by optimizing the concentration of specific small molecules and the culturing method. In order to purify the progenitors, we built a transgenic reporter construct with a small V3-specific regulatory region based on the enhancer of the *Nkx2-2* gene to express the surface marker CD14 and to allow magnetic-activated cell sorting (MACS) enrichment. V3 progenitors enriched with this method matured in culture to postmitotic *SIM1*^+^ V3-interneurons.

## Results

### Human embryonic stem cell differentiation can be steered towards development of V3 progenitors

**Figure 1:**
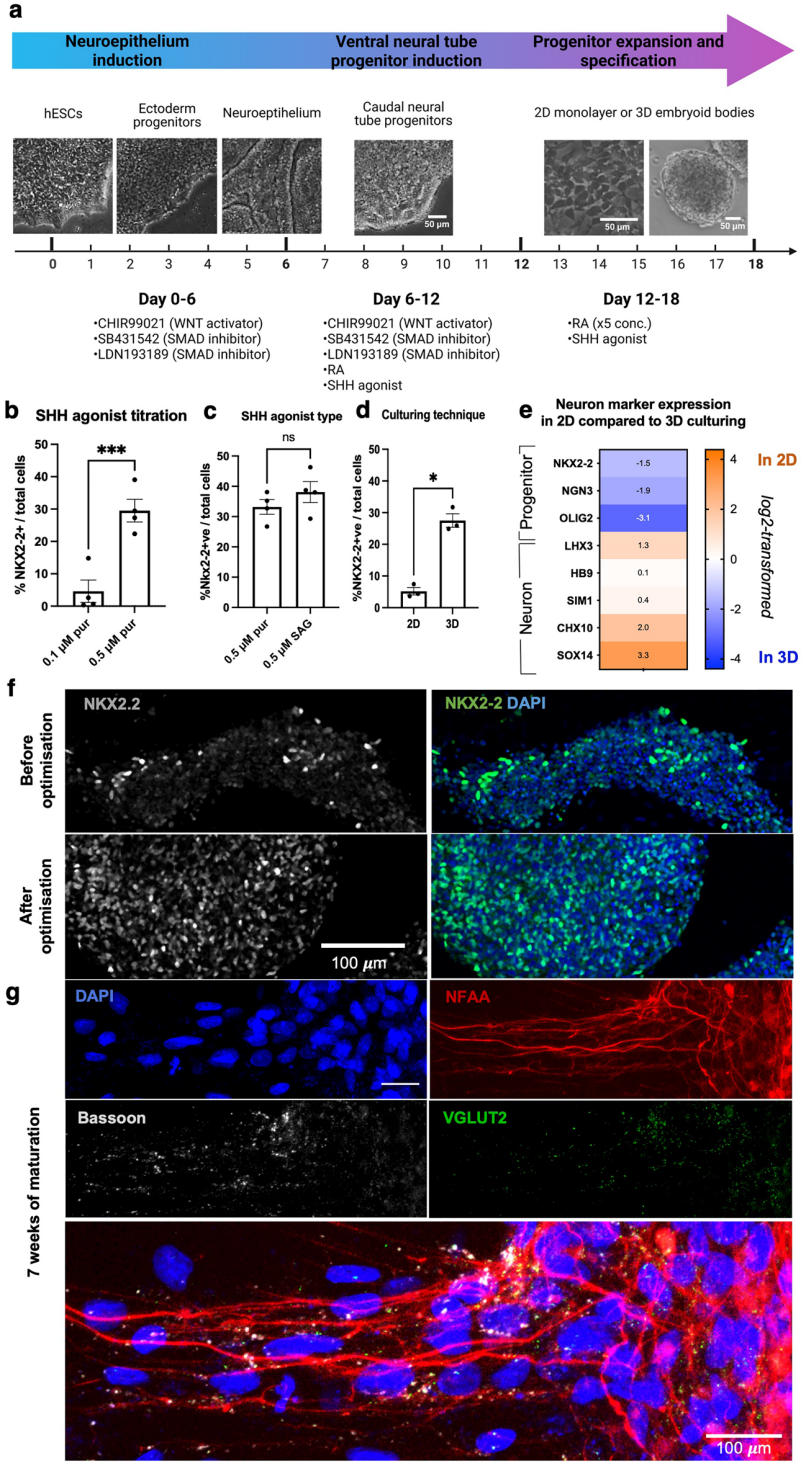
Optimisation of hESC differentiation towards NKX2-2^+^ V3 progenitors. Optimisation steps were analysed on d18, unless specified otherwise. (**a**) A timeline of differentiation up to day 18, with representative bright field images for each stage. Differentiation was performed in basal media (Supplementary Table S2) with molecules indicated in the diagram. Schematic has been created using a template^44^ from Biorender.com. (**b**) SHH agonist purmorphamine titration between d6–d18; 3D culture between d12-d18. Shown as mean ± s.e.m. (standard error of mean); n = 4. Two-tailed paired t-test, *p = 0.0003. (**c**) Comparison of the two SHH agonists between d6-d18; 3D culture between d12–d18. Performed in one differentiation, separate plates expressed as replicates. Shown as mean ± s.e.m; n = 4. Two-tailed paired t-test, p = 0.2983, n.s. SAG—smoothened agonist. (**d**) *2D*: d12 progenitors dissociated into a single cell 2D monolayer; *3D*: cells dissociated into clumps and plated as embryoid bodies in suspension; cultured in 0.5 μM purmorphamine*.* Shown as mean ± s.e.m.; n = 3. Two-tailed paired t-test, *p = 0.0159. (**e**) Relative gene expression by qRT-PCR, normalized to the housekeeping gene TATA box binding protein (TBP). Shown as a log2-transformed fold change of 2D- to 3D-cultured cells. Analysed on d25. (**f**) Images show V3 progenitors (NKX2-2^+^) and total cells (DAPI) derived using protocols before and after final optimisation. (**g**) Neural progenitors derived with the optimised protocol matured on a mESC-astrocytic monolayer expressing glial cell line-derived neurotrophic factor (GDNF) until d67. Presynaptic marker example—Bassoon, *NFAA* neurofilament associated antigen, *VGLUT2* vesicular glutamate transporter 2—glutamatergic interneuron marker.

Spinal neuronal progenitors can be derived from PSCs using directed differentiation, which mimics normal embryonic development. Since there was no previously established protocol for deriving V3 progenitors from human PSCs, we used a protocol suitable for the generation of spinal MNs^25,26^ as a starting point. To derive V3 progenitors, neuroepithelial identity was first induced for the first 6 days of culture combining inhibition of SMAD signaling and activation of WNT pathways^25^ (Fig. 1a). Sonic Hedgehog (SHH) and retinoic acid (RA) mediate the specification of ventral neural tube progenitors^27^, therefore, on day 6 of differentiation (d6), the induction media was supplemented with RA and an SHH agonist. Higher SHH agonist purmorphamine concentration (0.5 μM) led to a significant increase in the number of V3-NKX2-2^+^ progenitors compared to a lower concentration (0.1 μM)—28% and 1.6% of total cells, respectively (Fig. 1b). Prolonging SHH agonist incubation did not lead to significant changes—although a slightly earlier timepoint (d16) could be more optimal than a later timepoint (d20) (Supplementary Fig. 1a). Using a more potent Smoothened agonist (SAG) compared to purmorphamine (half maximal effective concentration (EC50) of 3 nM and 1.5 μM, respectively)^28^ led to a small but insignificant increase (Fig. 1c), indicating that the upper limit of SHH stimulation had been reached; 0.5 μM SAG was used in further experiments. RA concentration was titrated for both agonists, without substantial changes in either condition (Supplementary Fig. S1b).

In addition to molecular cues, we assessed if cell culturing methods which affect cell adhesion and density could impact the differentiation. When progenitors begin to form (ca. d12), cells can either be cultured in a 3D suspension, following dissociation into large embryoid bodies (*d* ~ 500 mm), or as a 2D monolayer (Fig. 1a). We observed a significantly lower percentage of NKX2-2^+^ cells in the 2D culture (5.2%), compared to 3D (27.5%) (Fig. 1d). Several other neuronal progenitor markers (NGN3 and OLIG2) showed a similar reduction in expression when cells were cultured as a 2D monolayer (Fig. 1e). In contrast, the post mitotic markers of V3 interneurons (SIM1, OLIG3), MNs (HB9) and V2a interneurons (CHX10, SOX14, LHX3) showed upregulated expression in the 2D format (Fig. 1e). Because our V3 progenitor purification strategy was based on NKX2-2 expression, we chose the 3D culture in further experiments.

Differentiated neural progenitors with the newly optimised protocol produced embryoid bodies with a dense NKX2-2^+^ nuclei distribution (Fig. 1f). When plated on mESC-derived astrocytes, after 7 weeks of maturation, cells extended neurites positive for a pre-synaptic marker Bassoon, as well as glutamatergic-specific VGLUT2 marker (Fig. 1g), suggesting maturation to glutamatergic V3 interneurons.

### V3 interneuron progenitors can be isolated using a transgenic surface marker

**Figure 2:**
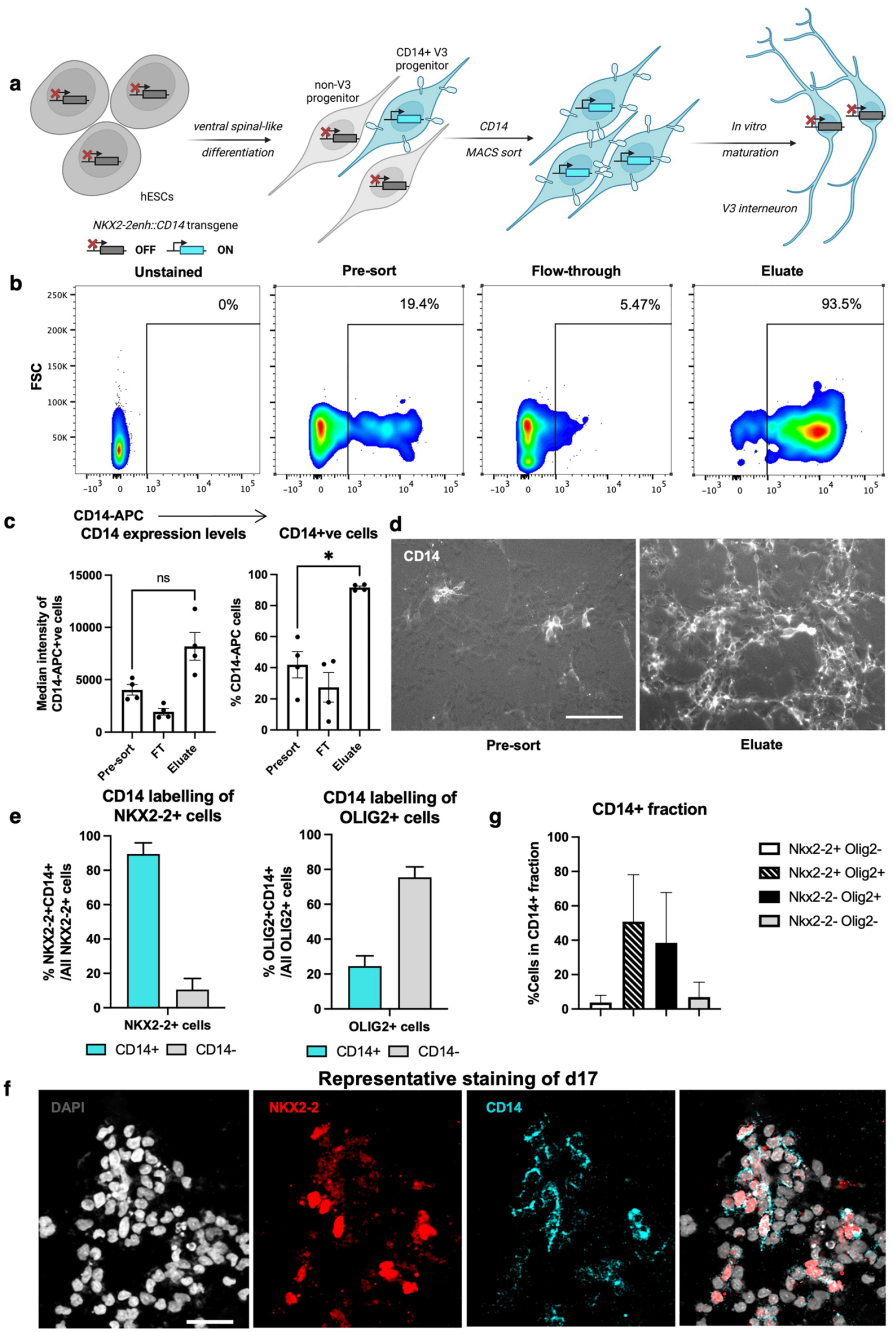
V3 progenitors can be purified using an in-built surface marker *Nkx2-2enh::CD14*. (**a**) A schematic for V3 progenitor purification strategy using an in-built surface marker *Nkx2-2enh::CD14.* The transgene inserted into the genome of an H9 hESC line should be transcribed in differentiated cells, in which *NKX2-2* enhancer is active. In the context of ventral spinal-like differentiation, these should be mainly V3 progenitors. (**b**) A representative example of CD14-APC FACS analysis of MACS sorting fractions, shown as contour plots, with percentage of CD14^+^ gated cells. 10,000 cells were analysed. (**c**) CD14 expression in MACS sorting fractions, either by median intensity of CD14-APC or the number of CD14^+^ cells, analysed using FACS on d25. Shown as mean ± s.e.m.; n = 4, two-tailed paired t-test, for CD14 expression levels n.s. p = 0.0738, for CD14^+^ cells: *p = 0.0108. (**d**) Overlay images of bright field and Cy5 filter (CD14-APC) of pre-sort and eluate fractions, taken 24 h after sorting, live stained with anti-CD14-APC antibody. Cells were plated at the same density. Scale bar—50 mm. (**e**) Differentiated unsorted progenitor cells containing *Nkx2-2enh:*:*CD14* construct stained for the transgenic CD14 marker and the endogenous NKX2-2 (left) and OLIG2 (right)*.* Shown as means ± s. d. (standard deviation) of 8 technical replicates. Analyzed on d17. (**f**) Representative images of CD14-APC live staining and fixed endogenous NKX2-2 protein staining below the graphs. Scale bar—20 µm. (**g**) Proportion of CD14^+^ and NKX2-2^+^ or CD14^+^ and OLIG2^+^ cells out of all CD14^+^ cells. Shown as means ± s.d. of 8 technical replicates.

To further enrich V3 progenitors, we designed a purification method^29^ based on V3-specific expression of the transgenic surface reporter human CD14 and subsequent isolation of labelled cells by magnetic-activated cell sorting (MACS) (Fig. 2a). To target reporter expression to V3 progenitor cells, we designed the transgene vector such that the CD14 open reading frame is under the control of *Nkx2-2* gene enhancer. This enhancer construct has been previously shown to restrict *Nkx2-2* expression to the V3 progenitor domain (p3) of the developing neural tube in mouse and chick^30^. It contains a Gli protein-activating site, as well as Tcf protein-repressing sites that help to induce the *Nkx2-2* expression in the V3 domain, and exclude it from the neighboring motor neuron progenitor (pMN) domain.

We incorporated the mouse *Nkx*2-2 enhancer (*Nkx2-2enh)* region combined with a minimal promoter (Supplementary Table S1)^30^ into the *Nkx2-2enh::CD14* construct (Supplementary Fig. S2), and inserted it into the AAVS1 locus in hESC genome by gene targeting^31^. Although this type of ectopic transgene insertion may not recapitulate the endogenous pattern of *Nkx2-2* gene expression, AAVS1 was chosen as a known locus to remain active upon hPSC differentiation into neurons^32^, which should ensure CD14 marker expression in V3 progenitors. Transgenic hESCs were differentiated, and labelled cells were isolated by MACS: CD14^+^ cells were significantly enriched after MACS sorting compared to the pre-sort population, as shown by FACS analysis (from 40 to 92%) (Fig. 2b,c) and immunostaining (Fig. 2d). To determine if the transgenic *Nkx2-2enh::CD14* marker confers specificity to V3 progenitors in the mix of differentiating cells, we co-labelled the CD14 with endogenous markers. Most of the NKX2-2-expressing V3 progenitors had the CD14 marker labelling associated to them (89% ± 6.5), whereas the majority of OLIG2^+^ cells were CD14-negative (75.5% ± 5.3) (Fig. 2e,f). When assessing the CD14^+^ cell populations, we found that CD14 labelling was associated with NKX2-2^+^ cells (54.6% of the total CD14 fraction) but also with NKX2-2^-^ OLIG2^+^ cells (38.4%) (Fig. 2g). In summary, the *Nkx2-2enh::CD14* construct labelled NKX2-2^+^ V3-like progenitors consistently, but we also observed ectopic expression in cells with a motor neuron progenitor phenotype. Although in mouse and chick, the same construct expression was restricted to the p3 domain only^30^, our findings indicate that in human models additional regulatory sequences could be necessary. We next investigated human-specific dynamics of NKX2-2, OLIG2 and *Nkx2-2enh::CD14* expression to understand if the construct could be used to separate V3 progenitors specifically.

### Human NKX2-2 expression specificity increases with time during differentiation

**Figure 3:**
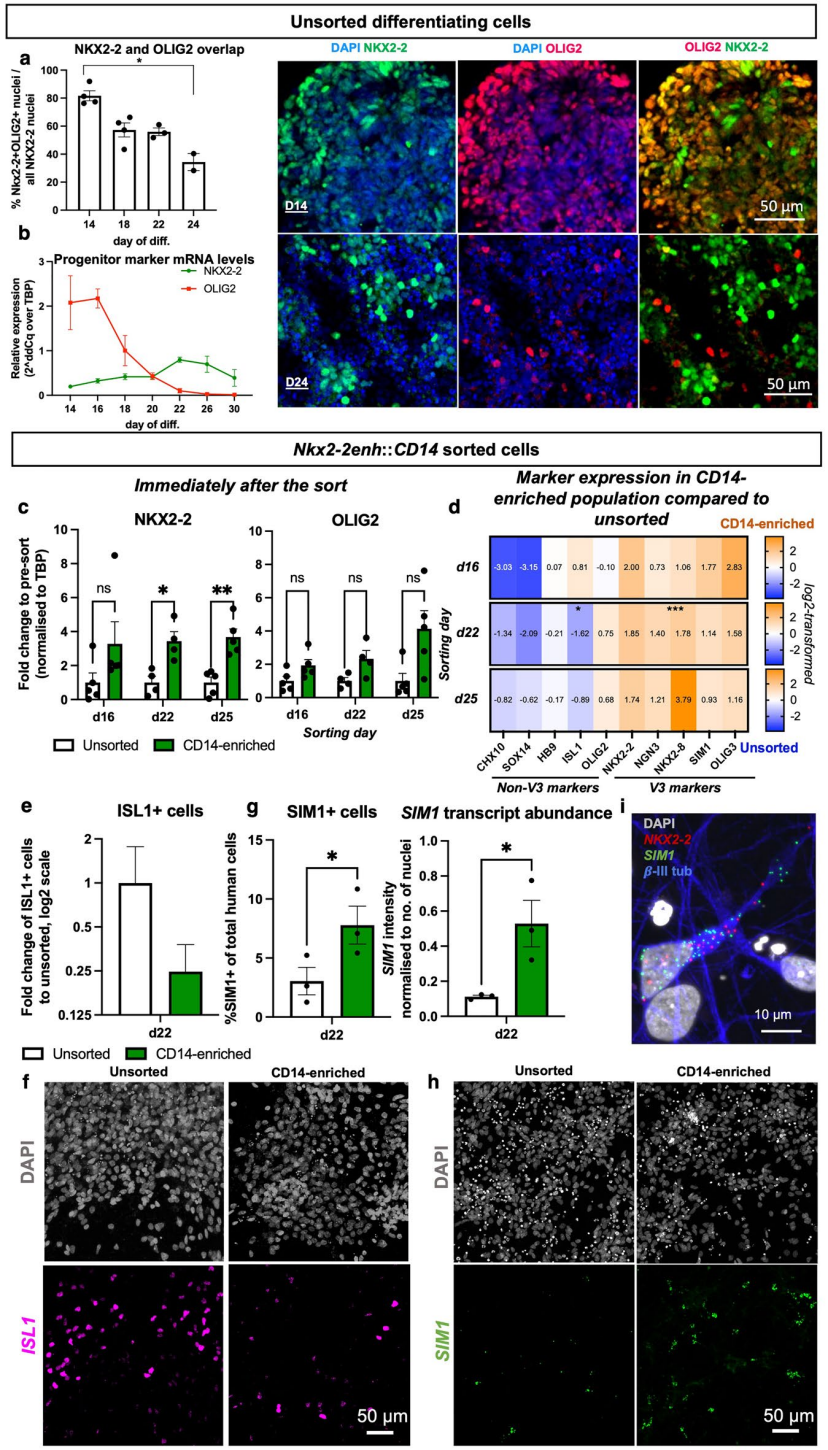
NKX2-2 overlap with OLIG2 declines with time in differentiating hESC-NPCs. (**a**) Developing unsorted embryoid bodies stained for NKX2-2, OLIG2 and DAPI (all nuclei). Quantification of the overlap between the two markers: NKX2-2+ OLIG2+ cells over total NKX2-2+ cells. Kruskall–Wallis with Dunn’s post hoc test, *p = 0.0182 for d14 vs. d26. Means ± s.e.m.; d14 & 18, n = 4; d22, n = 3; d24, n = 2. (**b**) Relative mRNA expression of NKX2-2 and OLIG2 in an unsorted differentiating culture. Means ± s.e.m., for *NKX2-2:* d16 & d20, n = 2; d14, 18, 22, 26 & 30, n = 3; for *OLIG2:* d16 & d20, n = 2; d14 & d30, n = 3; d18, d22 & d26, n = 4. (**c**) Cells sorted for CD14 marker on d16, d22 or d25 of differentiation, lysed immediately after the sort. Shown as a fold change to unsorted population of the respective timepoint. Mean ± s.e.m., n = 5; d22, n = 4; mixed model ANOVA with Šídák's multiple comparisons test: for NKX2-2, *p = 0.0419 for d22, **p = 0.0073 for d25 pre-sort vs. eluate. (**d**) Cells sorted for CD14 marker on d16, d22 and d25 of differentiation, lysed on d30. Values shown as a fold change of eluate to pre-sort samples. All gene expression normalized to TBP expression. Heatmap shows log2-transformed values. n = 3, except for CHX10 & SOX14, n = 2; multiple paired ratio t-tests with false discovery rate Q = 5%; for d22 ISL1 *p = 0.012, for d22 NKX2-8 ***p < 0.001 pre-sort vs. eluate. (**e**) Interneurons sorted on d22 were plated on a GDNF–expressing mESC-astrocyte monolayer at 5 to 1 ratio, respectively, with 10 ng/μl human recombinant brain-derived neurotrophic factor (BDNF) and neurotrophin-3 (NT-3); analysed on d28. ISL1^+^ cells over total cells (DAPI), expressed as a fold change to unsorted population. Shown as means ± s.d. of 8 technical replicates. (**f**) A representative example of ISL1 protein staining in unsorted and CD14-sorted on d22 cells. Analysed on d28. (**g**) *SIM1* stained using RNAscope Multiplex Fluorescent Assay kit. Shown as mean ± s.e.m.; n = 3, two-tailed paired *t*-test, *p = 0.04 for SIM1+ cells, and *p = 0.034 for *SIM1* transcript abundance. (**h**) A representative image used in the analysis performed in (**g**). (**i**) A representative image of *SIM1* and *NKX2-2* mRNA co-labelling with TUBB3 protein.

NKX2-2 is a specific developmental marker of V3 progenitors in the neural tube of chicken and mouse embryos^33,34^. However, in humans, it has recently been shown that NKX2-2 and the MN progenitor marker OLIG2 are co-expressed^25,35,36^. Although this overlap of expression is essential for oligodendrocyte development in the gliogenic stage of differentiation^37^, it was also observed in the neurogenic stages. Consistent with the earlier reports, we detected co-labelling of the two markers early in differentiation, with the extent of the overlap decreasing with the time in culture (Fig. 3a). In order to determine how the co-expression of *OLIG2* and *NKX2-2* markers affects the suitability of *Nkx2-2enh*:*:CD14* construct to label V3 progenitors specifically, we investigated three timepoints of in vitro development (Supplementary Fig. S1c): an early timepoint (d16) with a high *OLIG2* and a low *NKX2-2* expression (Fig. 3b); an intermediate timepoint (d22) with *NKX2-2* expression higher than *OLIG2*; and the latest timepoint embryoid bodies maintained high viability before becoming hypoxic (d25). When analyzed immediately after the sort, cells in the CD14-enriched eluate showed a significant three to fourfold upregulation of endogenous *NKX2-2* expression at mid (d22) and late (d25) sorting timepoints (Fig. 3c). *OLIG2* enrichment was also in the range of two to fourfold upregulation, although a lot more variable and statistically insignificant (Fig. 3c). Overall, the transcriptional separation between *NKX2-2* and *OLIG2* was not apparent when analysed immediately after sorting.

When sorted cells developed in culture up to d30, the timepoint of CD14-based cell enrichment became crucial for the V3 interneuron and MN separation: cells sorted on d22 showed an enrichment in V3 progenitor (*NKX2-2*, *NKX2-8*) and post-mitotic *(SIM1, OLIG3*) markers, and there was a significant depletion in the post-mitotic MN marker *ISL1* (Fig. 3d)*.* The fraction of ISL1^+^ MNs in the culture was also reduced (Fig. 3e,f). Cells sorted even later in development (d25) maintained the enrichment in V3 markers and the depletion in MN markers *ISL1* and *HB9*. In contrast, when sorted earlier in development, on d16, cells showed enrichment in both, V3 interneuron, as well as MN post mitotic markers (Fig. 3d). Depletion of V2a interneurons (*CHX10* and *SOX14* markers) was detected at all three sorting timepoints with the strongest depletion when sorted on d16.

When cells convert from V3 progenitors to post-mitotic interneurons, they upregulate the V3-specific post-mitotic marker SIM1. The sorted population maintained a significant enrichment of V3-identity neurons, as shown by RNAscope analysis of *SIM1* transcripts (Fig. 3g,h), and co-labeled with the neuronal marker tubulin-βIII (Fig. 3i). Many of the *SIM1-*labelling neurons also expressed the *NKX2-2* marker (Fig. 3i), suggesting that the full conversion and maturation in human cells could take longer, even in the presence of neuronal maturation-stimulating growth factors [brain-derived neurotrophic factor (BDNF) and neurotrophin-3 (NT-3)].

## Discussion

In this study, we report the successful enrichment of human PSC-derived V3 spinal interneurons and their progenitor population. We used human ESCs and found key parameters that played a role in NKX2-2^+^ V3 interneuron progenitor formation in a small molecule-driven in vitro differentiation. First, using an SHH agonist at a higher concentration was important in generating up to 40% of V3 progenitors. This is in agreement with observations that the p3 domain in the neural tube is the closest to the SHH-secreting floor plate, requiring the highest levels of SHH stimulation for their identity acquisition^38^. A second important feature was the culturing method: we observed that keeping cell differentiation in a 3D format (as embryoid bodies) maintained progenitor marker expression, whereas dissociation into a 2D monolayer showed a reduction in multiple progenitor markers and an upregulation in postmitotic markers. The sustained neural progenitor expansion in 3D cultures could be an important consideration for studies requiring a large number of progenitors, for example, for in vivo cell transplantations. Finally, after the protocol optimisation, cultures with V3 interneuron progenitors were dissociated and plated for 7 weeks, during which the cells extended neurites positive for glutamatergic subtype-specific (VGLUT2) and general neural maturation markers (Bassoon, Neurofilament-associated antigen). Although human neurons take a long time to mature, expression of glutamatergic synapses indicate that the cells could be used in in vitro functional assays, such as electrophysiological reads of neuronal activity and histological assessment of connectivity to other cell types.

Additionally, we developed a transgenic surface marker (*Nkx2-2enh:*:*CD14)* for MACS–based purification—a scalable and a cost-effective system—and we were able to recover highly enriched populations of CD14^+^ V3 neural progenitor cells. We showed that in human cells, there was an overlap of the NKX2-2^+^ V3 and the OLIG2^+^ MN progenitor marker expression; the transcriptional separation of these two lineage markers was investigated and determined to occur in later stages of neurogenesis. Therefore, when using *NKX2-2enh*:*:CD14* based MACS sorting, we found that sorting at later timepoints (d22 and d25) successfully reduces MN-associated markers, such as ISL1, from the culture. MACS-based sorting enriched V3 progenitors from ~ 40% in mixed differentiation cultures to > 90% in the MACS eluate fraction. Post-mitotic conversion and functional maturation of the human PSC-derived neurons is still a challenge within the field, partly due to limited research on their survival requirements, as well as slow development in a human model^16,39^. Using CD14-based MACS sort, we also observed an enrichment in *SIM1*^+^ postmitotic V3 interneurons. The conversion of V3 progenitors into post-mitotic interneurons has so far proven to be a challenge for in vitro cultures, however, our CD14-based MACS sort led to an enrichment in *SIM1*^+^ post-mitotic V3 interneurons as well. Longer culturing time with an optimal growth factor combination could improve this conversion further.

The derivation and cell sorting strategy for spinal V3 interneurons from hESCs reported in this study will facilitate the in vitro assembly of local spinal neural networks controlling locomotion from their constituent cellular elements, and support future projects in at least three areas of spinal cord research: first, transplantation of stem cell-derived neural grafts into injured spinal cord with the aim of restoring motor function; second, modeling of degeneration of locomotor circuits in neuromuscular diseases; and third, modeling of formation and maturation of neural networks during normal human spinal cord development.

## Methods

### Human embryonic stem cell maintenance and differentiation

The hESC H9 line^40^ was acquired from WiCell under a license from the steering committee for the UK Stem Cell Bank (No. SCS11-06). Cells were expanded on plates coated with laminin 521 (LN521, BioLamina) at 0.5 μg/cm^2^ in DPBS (with Ca^2+^/Mg^2+^) at 4 °C overnight. StemMACS iPS-Brew XF with iPS-Brew supplement (1x) (130-104-368, Miltenyi Biotec) was used for maintenance—with 10 µM of ROCK inhibitor Y-27632 when recovering cells after defrosting or splitting into single cells.

We used the human PSC differentiation protocol for human spinal MNs published before^25,26^ with minor modifications (Fig. 1a). Frozen aliquots of 1 × 10^6^ H9 cells were defrosted into two wells of a 6-well plate, expanded for 3 days to an 80% confluency and then transferred as colonies onto a 100 mm Matrigel-coated plate (Growth Factor–Reduced (GFR) Matrigel, 356238, Corning). Plates were pre-coated with GFR-Matrigel diluted 1:50 in DMEM (1–2 h at 25 °C or 4 °C overnight). Cells on laminin were dissociated as clumps using Versene for 3 min at 37 °C and handled carefully with a 10 ml stripette to retain large aggregates. The next day (d0), they were induced in basal medium (Supplementary Table S2) with small molecules 3 µM CHIR99021, 2 µM SB431542 and 0.2 µM LDN193189 until d6 (Fig. 1a). On d6, cells were split 1:3 by incubating with 1 mg/ml collagenase IV for 12 min at 37 °C onto Matrigel-coated plates in basal medium with 0.1 µM RA, 0.5 µM purmorphamine, 1 µM CHIR99021, 2 µM SB431542 and 0.2 µM LDN193189 for another 6 days. Smoothened agonist (SAG) was used at 0.5 µM instead of purmorphamine during optimisation. Embryoid body suspension culture was performed in non-treated culture 10 cm plates (430591, Corning).

### hESC-derived neuron maturation on astrocytes

mESC-derived astrocytes were used to support the maturation of hESC-differentiated V3 progenitors. mESCs were differentiated into astrocytes and purified from neurons using a previously described method^41^. The cell line contained the neurotrophic factor transgene *CAG::GDNF* and the astrocyte-specific *GFAP::CD14* reporter. Cells were differentiated for 12 days and then MACS-sorted for CD14-expressing cells^42^. Purified mESC-astrocytes were plated with hESC-neurons at 1:5 ratio, respectively, with 10 ng/μl human recombinant BDNF and NT-3.

### Generation of genetic constructs

The *Nkx2-2* enhancer^30^ was amplified from the mouse genomic bacterial artificial chromosome clone RP23-224O8 (BACPAC Resources Centre) using primers with overhangs compatible to pBluescript SK ( +) vector (Stratagene) cut with EcoRV enzyme (Supplementary Table S1). The amplified product was then inserted into the pBluescript SK ( +) vector cut with EcoRV using the InFusion HD cloning kit (638910, Clontech).

The final vector for *Nkx2-2* enhancer insertion, containing CD14 and flanking homologous sequences for gene targeting into the safe-harbour genomic locus AAVS1^43^, was prepared using the plasmid AAVS1-CAGGS-hrGFP ^43^ (#52344, Addgene) plasmid. First, the NotI restriction site in the backbone was removed, and the *CAGGS:*:GFP fragment was excised using SpeI and MluI sites (Supplementary Fig. S2). An artificial PCR product with multiple cloning site (MCS) was inserted between SpeI and MluI, and then NotI and PacI sites in the MCS region were used to insert the previously published Hb9 gene regulatory region^27^ driving the expression of *CD14* gene^29^ (*Hb9*:*:CD14* construct). The *Hb9* promoter was excised using NotI and AscI restriction enzymes, and the *Nkx2-2* enhancer, extracted from the pBS SK ( +) vector using NotI and AscI enzymes, was inserted. Finally, the β-globin minimal promoter was extracted from a plasmid purchased on Addgene (83897) using primers compatible to 3’ end of Nxk2-2 enhancer and 5’ end of CD14 gene (Supplementary Table S1) and inserted into the AAVS1 vector between the enhancer and the CD14 open reading frame using Gibson Assembly Master Mix (E2611S, NewEngland BioLabs).

### Generation of stable transgenic cell lines

Cells were pre-treated with 10 µM Y-27632 for 2 h prior transfection. Cells were dissociated for 3 min at 37 °C into a single-cell suspension using TrypLE Express (Invitrogen, 12605028) and resuspended in 100 µl of OptiMEM at 10 mln/ml density. They were mixed with 4 μg of the *Nkx2-2enh:CD14* in AAVS1 vector, together with 3 μg of left and 3 μg of right TALEN^43^. 2 mm electroporation cuvettes (Geneflow E6-0062) were used to electroporate with NEPA-21 electroporator (Nepagene) at the following settings: poring pulse 125 V, 5 ms pulse, 50 ms gap, two pulses, 10% decay (+ orientation); transfer pulse 20 V, 50 ms pulse, 50 ms gap, five pulses, 40% decay (± orientation). Cells were plated immediately after transfection with pre-warmed medium iPS Brew media and 10 µM Y-27632 at 47 k/cm^2^ to achieve single cell density. Y-27632 was removed once single cells had multiplied to several cell colony size and grown for 24 h before starting puromycin selection. Puromycin dihydrochloride was used at 0.5 µg/ml for 3–7 days until resistant colonies grew to 300–600 µm in diameter size. Colonies were transferred manually and expanded in 96 well plates.

After clonal expansion, cells were screened for construct insertion. Genomic DNA was extracted by lysing cells with QuickExtract DNA Extraction Solution (Epicentre QE09050) with 50 µl per well of a 96 well plate, incubating at 65 °C for 6 min, and 98 °C for 2 min. PCR reaction was run using Q5-HiFi Master Mix (NewEngland BioLabs M0492S). Two reactions for each clone were set up: insertion [Forward (5′-TCGACTTCCCCTCTTCCGATG-3′) and Reverse 1 (5′-GAGCCTAGGGCCGGGATTCTC-3′)] and wild type [Forward and Reverse 2 (5′-CTCAGGTTCTGGGAGAGGGTAG-3′)]^43^.

### Magnetic-activated cell sorting (MACS)

The *Nkx2-2enh::CD14* transgene-expressing cell fraction was enriched by MACS^29^ from in vitro differentiation cultures. Developing embryoid bodies were dissociated with Accumax at 37 °C at 400 rpm shaking for 10 min. Cells were labelled with anti-CD14-APC primary at 1:50 concentration (Supplementary Table S3), and after a wash, mouse anti-APC MicroBeads (130-090-855, Miltenyi) at 1:5 concentration in MACS buffer for 15 min each at 4 °C in a Miltenyi Biotec MACSmix Tube Rotator. Pre-sort, flow through and eluate fractions were analysed using a BD Fluorescence-activated cell sorting (FACS) Canto II machine (NIHR Guy’s and St. Thomas’ Biomedical Research Centre). DAPI was added at 1 µg/ml to exclude the dead cells just before the analysis. 10,000 cells were analysed using the FlowJo software (BD Biosciences). Live single cells were gated based on forward and side scatter (FSC and SSC) parameters, as well as DAPI absorbance. Unstained cells were used to gate for an APC-positive fraction.

### Fluorescent marker labeling and imaging

For fluorescent protein labelling, cells were grown as a 2D monolayer in 96 µClear well plates (655097, Greiner Bio-One) and fixed with 4% paraformaldehyde for 15 min at room temperature (RT) or as 3D-embryoid bodies that were collected and fixed in 4% PFA for 30 min at 4 °C. Embryoid bodies were then incubated in 30% sucrose solution in PBS until saturated and embedded in O.C.T. for cryosectioning at 18 µm thickness. Both monolayer cells and embryoid bodies were washed in PBS and then blocked in 3% BSA in 0.1% PBT (PBS with 0.1% Triton X-100) for 30 min at RT. Primary antibody (Supplementary Table S3) incubation was performed overnight at 4 °C; cells were washed three times in PBS and then incubated with secondary antibody (Supplementary Table S3) and DAPI (300 nM) for 1 h at RT. Fixed cells were imaged using confocal inverted laser scanning microscope Leica TCS SP8 and analysed using Fiji software (https://fiji.sc/). Nuclear marker-positive cells were automatically counted using the CellProfiler software (https://cellprofiler.org/).

Live cell staining was performed for 15 min at 37 °C in basal media (Supplementary Table S2). Live cell imaging was performed using Olympus IX71 epifluorescent microscope with U-HGLGPS light source and Cy5 filter.

### Quantitative real time polymerase chain reaction (qRT-PCR)

RNA was extracted using Invitrogen Purelink RNA Minikit or Microkit. Complementary DNA (cDNA) was prepared using GoScript Reverse Transcription System (Promega A5001). Gene expression was assessed using FAST SYBR Green MasterMix (Applied Biosystems 4385612) and primers at 200 nM concentration (Supplementary Table S4). Reaction was performed at the following settings: 95 °C 10 min; 40 cycles of 95 °C-30 s and 60 °C-1 min; 75 °C-10 s; 95 °C-10 s.

### RNAscope

Cells were fixed on d28 after culturing on 96 µClear well plates by incubating with 10% neutral buffered formalin solution for 20 min. After washing with PBS, they were dehydrated and rehydrated by incubating in varying ethanol concentration steps: 50%, 70% and 100% 5 min each at RT. A final 100% ethanol incubation step for 10 min was followed by rehydration at 70% and 50% ethanol for 2 min each. Cells were washed in PBS and then treated with RNAscope Hydrogen Peroxide for 10 min at RT, washed and then treated with 1:15 diluted Protease III for 10 min at RT. Cells were further incubated with diluted (1:50 in probe diluent) anti-human SIM1-C3 probe and undiluted anti-human NKX2-2-C1 probe for 2 h in HybEZ™ oven at 40 °C. The signal was amplified using Multiplex FL v1 Amp1 for 30 min, Amp2 for 15 min, Amp3 for 30 min and Amp4 for 15 min in the 40 °C oven. After each amplification step cells were washed twice with wash buffer (1×) for 2 min. Finally, RNAscope DAPI was applied for 30 s at RT and washed with PBS.

### Statistics

Statistical comparisons between two groups were performed using paired t-tests. All samples from the same differentiation attempt were considered “paired”. Samples from separate differentiations were considered as biological replicates and the statistical error was calculated as standard error of mean (s.e.m.). In the case of multiple comparisons, false discovery rate of Q = 5% was used. For multiple group comparison with low replicate number, non-parametric test was used, such as Kruskall-Wallis with Dunn’s post hoc test.

## Supplementary Information

Supplementary Information.

## Data Availability

The datasets generated during and/or analysed during the current study are available from the corresponding author on reasonable request.
