# Supplementary material for: Enrichment of human embryonic stem cell-derived V3 interneurons using an Nkx2-2 gene-specific reporter
Source: Sci Rep. 2023 Feb 3;13:2008. doi: 10.1038/s41598-023-29165-z (PMC9898512; doi:10.1038/s41598-023-29165-z)
Supplement: Supplementary file 1 — Supplementary Information. [file 41598_2023_29165_MOESM1_ESM.pdf]

Title: Enrichment of human embryonic stem cell-derived V3 interneurons using an *Nkx2-2* gene-specific reporter

Authors and addresses:

Ieva Berzanskyte* [1,2], Federica Riccio[1], Carolina Barcellos Machado[1], Elizabeth J Bradbury[2], Ivo Lieberam* [1]

1 Centre for Gene Therapy and Regenerative Medicine, Centre for Developmental Neurobiology and MRC Centre for Neurodevelopmental Disorders, King's College London, London, UK
2 The Wolfson Centre for Age-Related Diseases, King's College London, London, UK

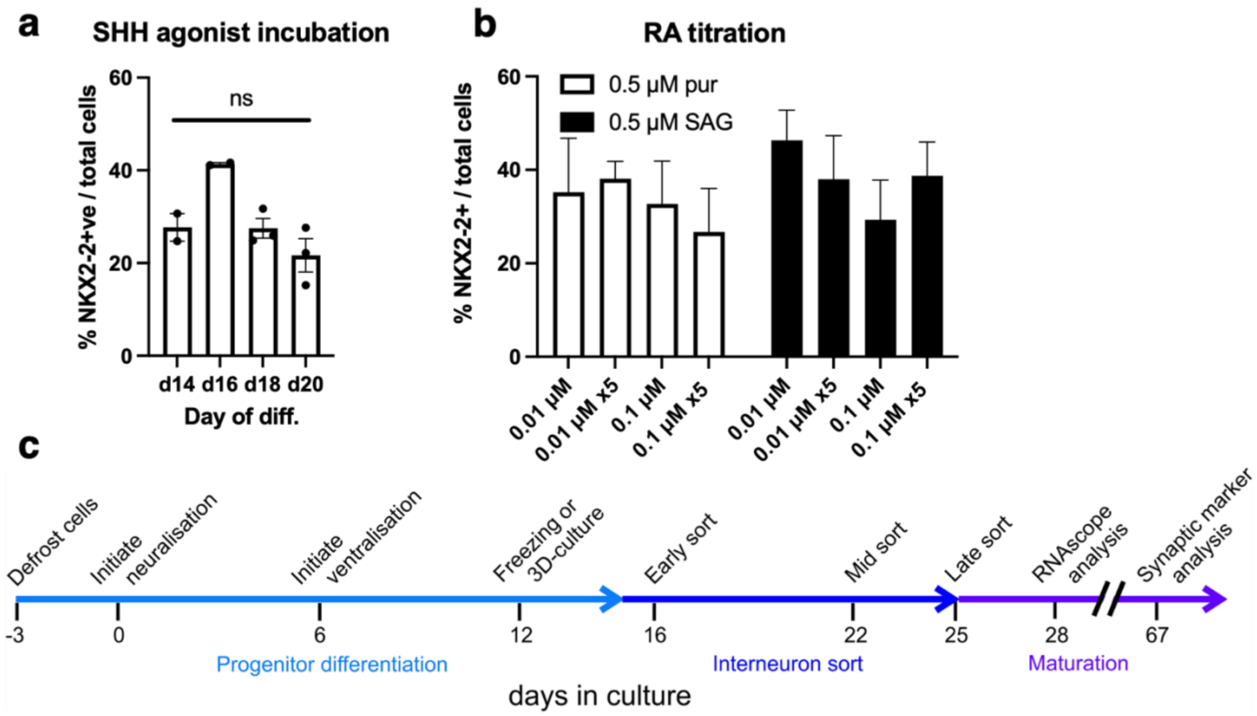

Supplementary Figure S1. A) Length of purmorphamine (0.5 μM) stimulation. Replicates are separate differentiations, n=2 for d14 and d16; n=3 for d18 and d20. Shown as mean±s.e.m. Kruskal-Wallis, p=0.11, n.s. SAG - Smoothened Agonist. B) RA titration for the two SHH agonists (purmorphamine and SAG). x-axis indicates RA concentration used from d6. If labelled with "x5", the concentration was increased 5-fold from d12 onwards (Fig. 1A). Performed in one differentiation, 4 separate wells expressed as technical replicates. Shown as mean±s.d. Analysed on d18. C) Experimental timeline. Progenitors were differentiated for 12 days from the initiation point (d0); interneuron sorting was performed during the expansion phase (d16 – 25); mature neuron analysis was performed on d28 and onwards - either plated on mESC-astrocytes or cultured alone. In both cases, NT-3, GDNF and BDNF were added to the culture.

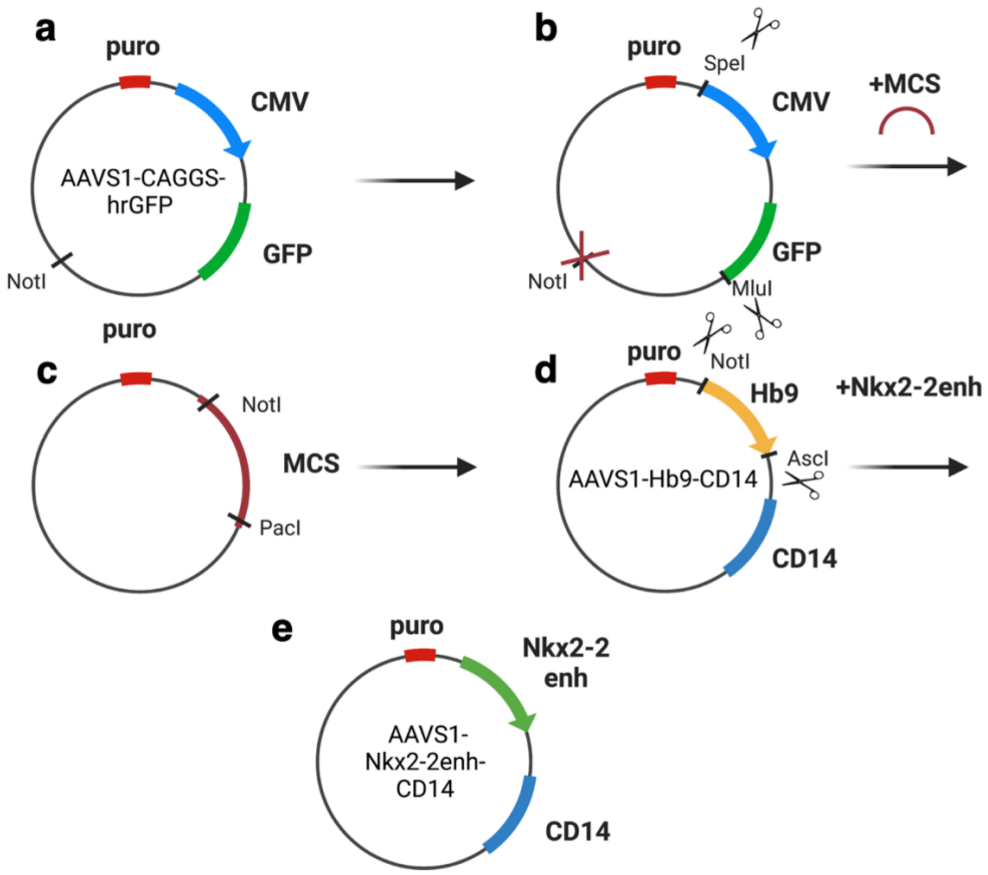

Supplementary Figure S2. Nkx2-2enh::CD14 insertion into AAVS1 vector. A) The original AAVS1 vector plasmid (#52344, Addgene). The NotI site was removed from the AAVS1 vector backbone, CAGGS::hrGFP was removed using SpeI and MluI restriction enzymes. B) An artificial PCR product containing multiple restriction enzyme sites was inserted. C) AAVS1 vector was linearised with NotI and PacI. D) A construct containing the Hb9 regulatory region and an open reading frame of CD14 surface marker (designed previously in[1]) were inserted. E) Using NotI and AscI sites, Hb9 regulatory region was replaced with Nkx2-2 enhancer (*Nkx2-2enh*). MCS – multiple cloning site. Not shown: the CD14 gene is flanked by a 5' splice substrate and a 3'polyAsignal. Illustration created with BioRender.com.

Supplementary Table S1: PCR primers used to extract *Nkx2-2* enhancer and β-globin minimal promoter

**Regulatory region**	**Forward primer (5'->3')**	**Reverse primer (5'->3')**
Nkx2-2 enhancer [2]	CGAGGTCGACGGTATCGATAAGCTTGATGCGGCCGCNNNN**TCTCCGGATCCCCAAGGAAA**	TGGATCCCCCGGGCTGCAGGAATTCGATTTAATTAAGCGAGGCGCGCCAATA**GTTGCCTTAGTCCAGGCGAA**
Minimal promoter[3,4]	CTAAGGCAACTATTGGCGCG**CCCGGGCTGGGCATAAAAGT**	GCAGACAGCGAGGCGCG**AGAAGCAAATGTAAGCAATAGAT**

Supplementary Table S2: Basal media composition

**Component**	**Company and catalogue no.**
DMEM/F-12 (1 part)	Gibco 21331020
Neurobasal (1 part)	Gibco 21103049
N2 supplement (0.5x)	Gibco 17502001
NeuroBrew21 (0.5x)	Miltenyi Biotec 130-093-566
L-glutamine (2mM)	Gibco 25030-024
Penicillin/streptomycin (1x)	Gibco 15140-122
NT-3 (10 ng/ml; for maturation phase)	Peprotech 450-03
GDNF (10 ng/ml; for maturation phase)	Peprotech 450-10
BDNF (10 ng/ml; for maturation phase)	Peprotech 450-02

Supplementary Table S3: Antibodies used for the experiments

**Antigen and dilution**	**Company and catalogue no.**	**Validation information**
NKX2-2, 1:50 or 4 μg/ml	Abcam 187375	3 publications: https://www.abcam.com/nkx22-antibody-nx2294-ab187375.html and chicken embryonic neural tube staining (**Suppl. Fig. 3**)
CD14-APC, 1:50 in 100 μl for 10^6 cells	Miltenyi REA599	By manufacturer: https://www.miltenyibiotec.com/GB-en/products/cd14-antibody-anti-human-reafinity-rea599.html#gref

OLIG2, 1:500 or ~0.28 μg/ml	Abcam 109186	94 publications: https://www.abcam.com/olig2-antibody-epr2673-ab109186.html
ISL-1, 1:1000 or ~0.75 μg/ml	Abcam ab20670	77 publications: https://www.abcam.com/islet-1-antibody-ab20670.html
GFAP, 1:1000	Millipore MAB360	List of references: https://www.merckmillipore.com/GB/en/product/Anti-Glial-Fibrillary-Acidic-Protein-Antibody-clone-GA5,MM_NF-MAB360#anchor_REF
SC101, 1:1000 or 0.5 μg/ml	Takara Y40400	110 citations: https://www.takarabio.com/products/antibodies-and-elisa/primary-antibodies-and-elisas-by-research-area/stem-cell-research-antibodies/stem-antibodies
TUBB3, 1:1000 or 0.5 μg/ml	R&D Systems MAB1195	172 citations: https://www.rndsystems.com/products/neuron-specific-beta-iii-tubulin-antibody-tuj-1_mab1195#product-citations
VGLUT2, 1:1000 or 1 μg/ml	Synaptic Systems 135403	14 immunocytochemistry references: https://sysy.com/product/135403
Bassoon, 1:500	Abcam ab82958	46 references: https://www.abcam.com/bassoonbsn-antibody-sap7f407-ab82958.html
NFAA, 1:1000	3A10 hybridoma line (DSHB)	74 references: https://dshb.biology.uiowa.edu/3A10

Supplementary Table S4: primers used for qRT-PCR

Gene	Forward 5'->3'	Reverse 5'->3'
NKX2-2	GTCCGGAGGAAGAGAACGAG	CCGTGCAGGGAGTACTGAAG
OLIG2	TCGCATCCAGATTTTCGGGT	GCAGAAAAAGGTCATCGGGC
NGN3	CTAAGAGCGAGTTGGCACTGA	GAGGTTGTGCATTCGATTGCG
LHX3	GGACGCTGACCTAGGAGGA	ATGAAGCGGTCCAGGATGTG
HB9	TGCCTAAGATGCCCGACTTC	AATCTTCACCTGGGTCTCGG
SIM1	GCCCGAGACACGATGAAAGA	CCAAGCCCTTCTGGAAAGACC
SOX14	GAACCCTTGCACTCCCTACC	ACAGAAGCTTCCATTCGGCA
CHX10	AGCGTCATGGCTGAGTATGG	AAGCCATCTTGAACCAGTAGCC
OLIG3	TGAAGATCAACGGACGCGAA	CCCCCATAGATCTCGCCAAC
TBP	GCCAGCTTCGGAGAGTTCTGGGATT	CGGGCACGAAGTGCAATGGTCTTTA

References

1. Paredes-Redondo, A. *et al.* Optogenetic modeling of human neuromuscular circuits in Duchenne muscular dystrophy with CRISPR and pharmacological corrections. *Sci. Adv.* **7**, eabi8787 (2021).
2. Lei, Q. *et al.* Wnt Signaling Inhibitors Regulate the Transcriptional Response to Morphogenetic Shh-Gli Signaling in the Neural Tube. *Dev. Cell* **11**, 325–337 (2006).
3. Yee, S.-P. & Rigby, P. W. The regulation of myogenin gene expression during the embryonic development of the mouse. *Genes Dev.* **7**, 1277–1289 (1993).

4. Dimidschstein, J. *et al.* A viral strategy for targeting and manipulating interneurons across vertebrate species. *Nat. Neurosci.* **19**, 1743–1749 (2016).
